# Monitoring helps services to reach the poor: the urban primary healthcare project in Bangladesh

**DOI:** 10.1186/1753-6561-6-S1-O10

**Published:** 2012-01-16

**Authors:** Kamal K Biswas, M Kabir, AB Sidique, Sharmin Mizan

**Affiliations:** 1 Urban Primary Health Care Project, Bangladesh

## Introduction

The Second Urban Primary Health Care Project (UPHCP-II) in Bangladesh started in 2005 with a mandate to extend every component of health services to at least 30% of the poor in catchment areas. Poor were identified through household survey and were provided with free service entitlement cards by the service delivery partners. UPHCP-II started service delivery in partnership with contracted non-government organisations (NGO) in 2005.

The Project maintains a robust health management information system with web-based data uploaded by the provider NGOs. In 2007, the project employed a third party (HLSP) for monitoring service delivery using specially designed tools called Integrated Supervisory Instruments. These tools measure performances of partner NGOs with pre set indicators including services to the poor.

In this paper we analyse this gap using a case of institutional delivery to assess the coverage of the poor over a period of 2005 to 2009.

## Methods

We took the data on institutional deliveries in 24 maternal hospitals form the health management information system of the UPHCP-II. Data quality was assured through regular monitoring with systemic approach. We did descriptive analysis to see the changes over the time.

## Results

We found that the percentage of poor women who delivered at 10 UPHCP-II clinics in Dhaka was 2.09% in 2005, 1.85% in 2006, and 5.07% in 2007. The achievement was far lower than the mandatory target of 30%. However after the systemic monitoring in place, we observed an increase in number of poor women using institutional delivery services from 5.7% in 2007 to 19.6% in 2008, and 28.75% in 2009. A similar trend of increase was observed in other city cooperation partnership areas also.

## Discussion

Pro-poor policy framework combined with regular monitoring with specific poverty-based indicators helps maternal health services to reach the poor.

